# Microbiological safety assessment of ready-to-eat cooked foods in the Addis Ababa School Feeding Program, Ethiopia

**DOI:** 10.1016/j.heliyon.2024.e38110

**Published:** 2024-09-19

**Authors:** Yihalem Tamiru, Abebe Ayelign, Afework Mulugeta, Samson Gebremedhin

**Affiliations:** a Center for Food Science and Nutrition, Addis Ababa University, Addis Ababa, Ethiopia; b Department of Public Health, College of Health Sciences, Mekelle University, Mekelle, Ethiopia; c School of Public Health, College of Health Sciences, Addis Ababa University, Addis Ababa, Ethiopia

**Keywords:** Drinking water quality, Food safety, Microbiological quality, School feeding program

## Abstract

This investigation assessed microbial contamination indicators in RTE school meals and drinking water in the Addis Ababa SFP, Ethiopia. Samples were collected from 18 primary school kitchens in March and April 2024. Microbiological analysis was performed on 37 cooked food samples and 18 drinking water samples using ISO and NMKL guidelines. The microbiological investigation of RTE prepared meal samples revealed an overall acceptable level of quality and safety. However, concerns were identified. Yeasts and molds surpassed reference standards in 78.4 % of samples (>10^2^ cfu/ml), *E. coli* exceeded standards in 10.8 % of samples (>10^2^ cfu/ml), and *S. aureus* counts exceeded limits in 5.4 % of samples (10^3^ -10^4^). Cooked rice the highest microbiological counts, especially of *E. coli and S. aureus.* Approximately 14.4 % of food samples were unsatisfactory, showing contamination from *E. coli, S. aureus,* and yeasts and molds.

Regarding drinking water, the non-potable percentage in drinking water was 23.4 %, raising concerns about APC microbial count, *TC,* and *FC.* In particular, 72 % of the drinking water samples surpassed the APC criteria (>100 cfu/ml), 16 % exceeded the *TC* standard (>1 cfu/ml), and 5.5 % exceeded the *FC* threshold. The microbiological quality of meals served through the Addis Ababa SFP generally met established standards. However, some food samples exceeded the permitted limits, indicating hygiene difficulties. Therefore, stringent premises and personal hygiene measures must be implemented to safeguard their safety and well-being of the school children.

## Highlights

• RTE school meals in Addis Ababa, Ethiopia showed acceptable quality in microbiological analysis.
• Drinking water samples in Addis Ababa: concerns over microbial count, total coliforms, fecal coliforms; 23.4% samples non-potable.
• Cooked rice: highest microbial counts, especially *E. coli and S. aureus.*

## Acronyms and abbreviations

AAEB: Addis Ababa Education Bureau
AAU: Addis Ababa University
APC: Aerobic Plate Count
BGBB: Brilliant Green Bile Broth
CFU: Colony-Forming Unit
CFU/ml: Colony Forming Units per Milliliter
CNS: College of Natural and Sciences
EPHI: Ethiopian Public Health Institute
FC: Fecal coliforms
FSNRD: Food Science and Nutrition Research Directorate
FSQ: Food Safety and Quality
FSQS: Food safety and Quality Strategy
IDRC: International Development Research Center
IRB: Institutional Review Board
ISO: International Organization for Standardization
LMICs: Low- and Middle-Income Countries
MoA: Ministry of Agriculture
NMKL: Nordic-Baltic Committee on Food Analysis
QSAE: Quality and Standards Authority of Ethiopia
RTE: Ready-to-Eat
SFPs: School Feeding Programs
SWOC: Strengths, Weaknesses, Opportunities and Challenge
TC: Total Coliform
TVC: Total Viable Count
VRBA: Violet Red Bile Agar

## 1 Introduction

School feeding programs play a vital role in national social protection systems globally, offering anticipated benefits such as enhancing educational capabilities, addressing hunger, reducing malnutrition, improving school attendance, academic performance, and promoting gender equity in education [[1], [2], [3]]. These programs, often viewed as government support initiatives, significantly impact both education and children's health [4], emerging as crucial strategies in many countries to aid socially vulnerable individuals amidst economic hardships [4].

The global presence of foodborne pathogens in RTE foods poses significant health risks, particularly in LMICs where these foods are prevalent. Inadequate hygiene practices among vendors in these regions heighten the risk of microbial contamination, necessitating improved food safety regulations and practices to safeguard public health [[5], [6], [7]]. RTE foods, widely consumed by school-aged children and the working class in LMICs, significantly impact dietary intake. A decade-long study on the microbiological safety of RTE foods in LMICs emphasizes their critical role as essential dietary sources, despite risks arising from poor hygiene, poverty, limited health knowledge, and lax regulations [5].

Contaminated drinking water is a critical global health issue, leading to severe illnesses and fatalities, especially in developing regions, where poor water quality not only hampers economic progress but also jeopardizes the lives of millions [8]. Approximately 40 % of the African population lacks access to improved water supply and sanitation [9]. The global safety of drinking water is compromised by contaminants from various sources, underscoring the pivotal role of microbiological standards in ensuring pathogen-free and unpolluted water [8]. While access to clean water is prevalent in Europe and North America, many developing nations struggle with inadequate water and sanitation infrastructure, resulting in widespread waterborne infections [8,10]. The scarcity of safe drinking water correlates closely with increased morbidity and mortality from diseases associated with poor sanitation practices [8,11].

Despite the widespread implementation of SFPs globally, coverage varies significantly, with disparities between high-income and low-income countries [12]. Ensuring food safety remains a global challenge, affecting both developed and developing nations, with significant impact on public health and economic progress [13]. Ethiopia faces challenges in its food safety system, including gaps in legal frameworks, surveillance, laboratory services, and coordination among food safety organizations [13].

The QSAE plays a crucial role in ensuring food safety through certification, inspection, and testing, following international standards [13]. However, Ethiopia's food safety system faces hurdles due to a less developed food infrastructure, population growth, resource constraints, and hygiene challenges [13], key obstacles include surveillance of foodborne diseases, coordination among food safety entities, laboratory services, and fostering public-private partnerships [13].

Ethiopia's SFP, operational for over 30 years, is expanding and integrating with regional agricultural production [14]. The government is actively transforming the agricultural sector and SFP through effective policies [14]. With 26.8 million school-age children enrolled in pre-primary and primary education, Ethiopia launched a large-scale SFP in Addis Ababa in February 2019 to combat hunger [15,16]. This program aims to destigmatize school meals [17,18]. In the academic year 2022/23, government-sponsored SFPs benefited over 6 million schoolchildren, with the number of beneficiaries in Addis Ababa city administration (over 700,000) [16]. Moreover, the program has created employment opportunities for 10,120 mothers involved in food supply, cooking, and serving [19,20] However, ensuring the microbiological quality and safety of meals is critical to prevent foodborne illnesses among students. Despite this, concerns have been raised regarding food safety, encompassing ingredient quality, food items, and the hygienic conditions during meal preparation for students [21].

The microbiological quality of food reflects its microbial contamination level, with higher contamination indicating poor food storage and handling practices that increase disease transmission risks [22,23]. Bacterial counts in prepared food and water are crucial for assessing food safety and quality, shedding light on the hygiene standards maintained by food handlers during food preparation. Food and water are commonly recognized as vehicles for microbial disease transmission, including those induced by coliforms [23,24]. Recent concerns underscore the risks of microbial contamination in ready-to-eat meals, especially prevalent in high-volume settings like SFPs. The involvement of multiple individuals in large-scale food preparation [25,26], as seen in SFPs, increases the likelihood of contamination in the final product [25].

Food contamination risks are heightened in large-scale cooking settings, leading to foodborne diseases with significant health and economic impacts, particularly in developing nations [25,26]. RTE foods a common carrier of foodborne illnesses, contributes significantly to global outbreaks due to potential microbial contamination, posing grave health risks [6,7]. Also, poor hygiene practices during food preparation are key contributors to the prevalence of foodborne diseases [27].

Unintended contamination within food processes can act as a catalyst for foodborne disease outbreaks, exerting a profound impact on consumer health and national economies, particularly in developing regions [25,28]. This global challenge poses a substantial risk, with an estimated 582 million cases and 420,000 fatalities reported annually, with Africa shouldering a significant burden [29]. Notably, microbial food contamination stands as a pressing issue in global public health [30] A notable proportion of global outbreaks linked to educational settings are foodborne, accounting for around 45 % of incidents [4,31,32]. In the Republic of Korea, 47 % of documented cases of foodborne diseases were associated with schools, while Japan witnessed11,826 cases and 12 fatalities stemming from *E. coli* 0157:H7 infections in schools within the initial seven months of 1996 [33,34]. In Brazil, 11.6 % of reported cases in 2005 were related to school catering services [33,34]. An outbreak in South Wales, Great Britain, in 2005 affected 157 school children with *E. coli*, traced back to cooked sliced meats supplied to schools [33,34]. Similarly, France documented 544 adolescents affected by Salmonella food poisoning in 2010 [33,34]. Instances of foodborne illnesses have also been reported in Egypt, Cambodia, and India, where a considerable number of children fell ill or suffered from food-related ailments after consuming school meals [33,34].

A research conducted at Jashore University of Science and Technology in Bangladesh has highlighted heightened manganese levels, underscoring the indispensable significance of clean water for human well-being [35]. On a global scale, 884 million individuals lack access to safe drinking water, with regions such as Bangladesh facing formidable challenges in this regard [36]. In Africa, 40 % of the population grapples with inadequate water supply and sanitation facilities. Investigations carried out in Lesotho, Addis Ababa City in Ethiopia, Dire Dawa, Jimma, and North Gondar have revealed widespread bacterial contamination in water sources, indicating prevalent hygiene and quality issues [[37], [38], [39], [40], [41], [42]]. The persistence of waterborne diseases can be attributed to microbial pollutants, necessitating the implementation of heightened water safety protocols [37,43]. Contaminants, arising from fecal matter and infrastructural deficiencies, pose threats to piped water systems [37].

Children, especially those within educational institutions, are notably vulnerable to severe health complications induced by pathogenic bacteria present in tainted food sources [44,45]. The assurance of food safety stands as a pivotal endeavor in shielding individuals from illnesses and associated hazards, given the fundamental role that food plays in human health [46]. Nevertheless, the school setting poses unique challenges concerning disease transmission, with the potential for pathogenic microorganisms to contaminate food and water sources [44,47,48]. Bacteria, viruses, and parasites can endanger consumer health through contamination during production, handling, and storage. Evaluating microbiological quality is crucial to enforce safety measures for student meals.

Pathogens such as bacteria, viruses, and parasites pose a significant threat to consumer health, with contamination occurring at various stages including production, handling, and storage. The assessment of microbiological quality is essential to uphold safety standards for student meals. Microbiological contamination serves as a critical parameter that demands scrutiny to guarantee the safety of food prepared within catering systems [49]. Despite the presence of regulatory frameworks, school catering services frequently struggle to meet the required safety protocols during meal preparation. The oversight of specific sanitation guidelines across different phases of food handling jeopardizes the safety of students' meals [50].

Previous studies have emphasized the importance of monitoring and assessing the microbiological quality of RTE foods in SFPs to identify potential risks and prevent foodborne outbreaks. Risk factors such as cross-contamination, improper storage, inadequate cooking, and poor cleaning procedures have been identified as critical [49,51]. Several school-related outbreaks have been associated with food contamination resulting from unwell [52,53] or or unhygienic food handlers [52,[54], [55], [56]]. Indicator microorganisms are instrumental in routine food safety evaluations, aiding in appraising the hygiene practices of staff in food establishments [57,58] These microorganisms serve as essential tools for detecting and measuring the level of food contamination, with commonly utilized indicators encompassing TVC, *TC, E. coli,* and *S. aureus* [58,59]. Given that food provides an optimal environment for microbial proliferation, the risk of spoilage and foodborne illnesses is heightened [28]. Institutions like school cafeterias, which cater to large populations, face increased susceptibility to foodborne illnesses due to the extensive scale of food preparation, thereby escalating the potential for contamination [60,61].

Within Addis Ababa, the school kitchens participating in the homegrown feeding program offer student's meals like bread, rice, and injera with lentil-based sauce, served without reheating, yet information concerning the microbial safety of these offerings remains scarce. This study focuses on evaluating the microbial safety and quality of meals and drinking water provided in the school feeding program in Addis Ababa. By examining RTE cooked meals and water, the research aims to enhance food safety protocols and protect student well-being by identifying areas for improvement to ensure health and safety.

## 2 Materials and methods

### 2.1 Study area

Addis Ababa, the country's largest city, has significant political, economic, and symbolic importance [62]. By 2036, its population is projected to exceed 5 million, according to the central statistical agency [62]. The city government launched a large-scale SFP in Addis Ababa in February 2019 to combat hunger [15,16]. Currently, the school feeding program is implemented in all 264 public primary schools across 11 sub-cities, benefiting approximately 700,000 students [16]. The Addis Ababa SFP offered two school meals per day, comprising breakfast and lunch, throughout the week [63].

### 2.2 Study design and period

A school based cross-sectional study design was used to undertake this research work from March to April 15, 2024 in Addis Ababa, Ethiopia.

### 2.3 Inclusion and exclusion criteria

The study included RTE foods such as injera with sauces, rice, and bread regularly consumed by students in the school feeding program were included. Food samples that were inaccessible during the data collection process and any leftover foods from the selected sample were excluded from the study.

### 2.4 Sample size determination

To ensure comprehensive analysis and representative results, by the principle of rule of thumb (15–30 %) in the study area around eighteen public primary schools have been selected where the large number of students serve and cooked foods were presented in large. A structured sampling approach was employed. Three distinct types of food samples, including injera with sauce, rice, and bread, were collected per school due to their frequent consumption by students as part of the school feeding program. From each of the eighteen selected public primary schools, a maximum of three food types comprising two cooked food samples and one drinking water sample were gathered based on their availability. These samples underwent meticulous laboratory analysis to assess their microbiological quality and safety.

#### 2.4.1 Sample collection and handling procedures for food samples

In this study, 55 samples were collected for analysis, comprising RTE cooked food samples (n = 37) and drinking water samples (n = 18). The RTE cooked food samples were further classified into three groups: 16 samples of injera with lentil sauce, 14 samples of rice, and 7 samples of bread. The collection took place from March to April 15, 2024, at selected public primary schools. Sampling occurred on weekdays between 11:30 a.m. and 12:30 p.m., coinciding with designated serving times and based on availability. Each food type, amounting to approximately 250 g, was aseptically collected during each sampling session. These samples were carefully stored in sterilized plastic stomacher bags labeled with unique codes, subject names, and food types. The bags were sealed securely and kept below 4 °C in a cooler with ice for preservation. Subsequently, all samples were transported to the EPHI food microbiological laboratory in containers with ice. Upon reaching the laboratory, the samples were promptly analyzed, ensuring that each sample was processed on the same day it was received.

### 2.5 Microbiological indicators and quality criteria for food safety

#### 2.5.1 Microbiological indicators for food safety

In this study, we utilized common microbiological indicators to assess food safety in the Addis Ababa SFP. The indicators used were APC, *TC*, *E. coli,* and *S. aureus*. APC measures the overall microbial load in a food sample. Total coliforms indicate general hygiene and potential pathogen presence. *E. coli* signifies fecal contamination and potential pathogenicity. *S. aureus* can produce toxins causing foodborne illness and is linked to poor food handling and hygiene. Yeast and molds, while not direct safety indicators, are monitored for their impact on food quality and spoilage.

#### 2.5.2 Microbiological analysis parameters and quality criteria for cooked foods

We followed Codex Alimentarius recommendations for cooked foods to determine the analysis parameters for each sample group. These parameters included APC, *TC, E. coli, S. aureus,* yeast, and molds. To conduct the microbial analysis, we homogenized 25 g of each food sample with 225 ml of sterile buffered peptone water using a Bag Mixer for 2 min at speed 6. Serial dilutions were prepared using sterile buffered peptone water, and duplicate plates were made for each sample at each dilution following ISO 6887–1:2017 standard methods. Microbial counts were reported as CFU/ml. We assessed a total of 55 food samples, consisting of 37 food samples and 18 drinking water samples, to evaluate the microbiological quality and safety of RTE foods. This analysis involved detecting microbial indicators and pathogens, including APC, *TC, E. coli, S. aureus,* yeast, and molds.

Quality criteria were defined for each parameter to evaluate the microbiological quality. The criteria included APC <10^6^ cfu/ml, *TC* < 10^2^ cfu/ml, *FC* < 10^2^ cfu/ml, *E. coli* < 3 cfu/ml, *S. aureus* <10^2^ cfu/ml, and yeasts and molds < 10^2^ cfu/ml. The specific microbial parameters for water were evaluated, including APC <100 cfu/ml, TC < 1 cfu/ml, *FC* < 1 cfu/ml, and *E. coli* < 1 cfu/ml. These criteria were tailored to each food item and its preparation processes (Table 1).

**Table 1:** Reference standards for microbiological quality of RTE food and drinking water [[64], [65], [66], [67], [68], [69], [70], [71]].

Standards limit for RTE cooked food samples			
	Satisfactory	Marginal	Unsatisfactory
Indicator organisms	Guideline limit (cfu/ml) for ready-to-eat food		
APC	<10^6^ cfu/ml	10^6^-10^7^ cfu/ml	≥10^7^ cfu/ml
*TC*	<10^2^ cfu/ml	10^2^-10^4^ cfu/ml	≥10^4^ cfu/ml
*FC*	<10^2^ cfu/ml	10^2^-10^4^ cfu/ml	≥10^4^ cfu/ml
*E. coli*	<3 cfu/ml	3-100 cfu/ml	≥100 cfu/ml
*Staphylococcus count*	<10^2^ cfu/ml	10^2^-10^3^ cfu/ml	10^3^-10^4^ cfu/ml
Yeast and mold count	< 10^2^ cfu/ml		>10^2^ cfu/ml
Standards limit (cfu/ml) for drinking water samples			
Indicator organisms	Potable		Not-potable
APC	<100 cfu/ml		>100 cfu/ml
*TC*	<1 cfu/ml		≥1 cfu/ml
*FC*	<1 cfu/ml		≥1 cfu/ml
*E. coli*	<1 cfu/ml		≥1 cfu/ml

#### 2.5.3 Sample processing and preparation for analysis

Aseptically, 25 g of foods were weighed into sterile stomacher bags, blended with 225 ml BPW, and homogenized in stomacher for 2 min. Tenfold dilutions of homogenate were carried out with sterile peptone water (0.1 %; model 400, Seward Medical, London, UK) [29,72,73]. The analysis was performed on the same day as sample collection to ensure timely and accurate results.

#### 2.5.4 Aerobic plate count determination

Aerobic viable cells were counted using plate count agar (PCA, Difco Co., Detroit, MI, USA) and an automatic spiral plater (Spiral system, Model DU2, Cincinnati, OH, USA) The agar plates were incubated at 37 °C for 48 h [73]. After incubation, visible colonies were counted. Plates with colony counts ranging from 25 to 250 were selected for APC enumeration. The APC was calculated using the dilution factor and colony counts, and the results were expressed as CFU/mL or CFU/g [73].

#### 2.5.5 Enumeration of *E. coli* and coliform

Chromocult coliform agar (CCA, Merck Co.,Darmstsdt, Germany) was used to enumerate *E. coli* and coliform following the manufacturer's instructions [74]. Dilutions were plated and incubated at 37 °C for 24–48 h. Purplish red colonies were identified as coliform, while blue colonies were identified as *E. coli* [73,75]

#### 2.5.6 Enumeration of *S. aureus*

A 0.1 ml volume of the sample was spread evenly on Mannitol Salt Agar obtained from (HiMedia Laboratories Pvt. Ltd in Mumbai, India). The agar plates were incubated at 37 °C for 48 h to allow colony formation [73]. The *S. aureus* count was determined by observing typical colonies on the plates and conducting a positive coagulase test. Typical black colonies were further confirmed using rabbit plasma medium, which detects the presence of coagulase, an enzyme responsible for clot formation. The combination of enumeration on Mannitol Salt Agar and the positive coagulase test provided the *S. aureus* count [76]**.**

#### 2.5.7 Enumeration of TC

One milliliter of the food homogenate from each dilution was transferred to sterile Petri dishes. Then, 5 ml of molten Tryptone Soya Agar obtained from (HiMedia Laboratories plc in Mumbai, India) (kept at 45 °C) was poured into each dish. The dishes were incubated at 37 °C for 48 h, followed by further incubation at 35 °C ± 0.5 °C for 24–48 h with the agar plates inverted [75].

#### 2.5.8 Confirmation of TC

Selected colonies were inoculated into BGBB obtained from (Oxoid LTD in Basingstoke, Hampshire, England). The broth was incubated at 37 °C for 48 h, and positive coliform results were determined by examining gas production and turbidity after incubation [75].

#### 2.5.9 Enumeration of yeast and mold

A 0.1 ml portion of the food homogenate was spread onto Rose-Bengal chloramphenicol Agar obtained from (Micromix Pvt. Ltd in Verna, India). The agar plates were incubated at 25 °C for 5 days. Yeasts were identified by their creamy and white appearance, while molds were distinguished by their velvety texture and various colors [77].

#### 2.5.10 Sampling of drinking water

Water samples (n = 18) were collected from each selected school from March and April 15, 2024. The tap outlets were disinfected with 80 % alcohol, followed by the addition of 0.5 ml of sodium thiosulphate solution in the sterilized bottle to neutralize residual chlorine. Before sampling, the water was allowed to run for approximately 3 min. About 250 ml of water were aseptically collected in sterilized plastic bottles. The samples were transported with ice container to the EPHI food microbiology laboratory for analysis. The indicators analyzed included coliforms, *E. coli,* and aerobic mesophilic bacteria. Results were expressed as CFU/ml of water, and the samples were analyzed on the same day as collection.

#### 2.5.11 Aerobic plate count

One milliliter of the water sample was pipetted into each dish, ensuring thorough mixing for homogeneity. Standard plate count agar was melted and poured into each dish, covering the entire surface. The dishes were left undisturbed for 15 min to allow the agar to solidify. Inverted petri dishes were sealed in a plastic bag to create an appropriate incubation environment and placed in an incubator set at 35 ± 0.5 °C for 48 ± 3 h. After incubation, visible colonies on the solidified agar were counted using a colony counter. The colony counts were recorded, and the APC was calculated based on the number of colonies and the dilution factor. The results were compared to regulatory guidelines or established standards for drinking water quality [78,79].

#### 2.5.12 Determining coliform and TC using membrane filtration

Microbiological analyses were performed using the membrane filtration technique according to ISO protocols, for the detection of *E. coli*. Water samples of 250 ml each were filtered through a pore size 0.45 μm and 47 mm in diameter for all organisms. The membranes were placed in each Petri dish filled with a specific medium. The filters were placed on Tryptone Soya Agar plates for *TC* detection and VRBA plates for thermos-tolerant *E. coli* detection [80]. Incubation of the plates occurred at specific temperatures for 48 h: 37 °C for Tryptone Soya Agar plates and 44 °C for VRBA plates. After incubation, the plates were examined for characteristic coliform colonies. Representative colonies were selected and inoculated into brilliant green lactose bile broth, followed by incubation at 37 °C for 48 h to assess gas production and *confirm* the presence of *coliform* bacteria. Positive tubes were transferred to EC broth and subjected to the indole test after incubating at 44.5 °C for 48 h to confirm suspected *E. coli* [80].

For specific *E. coli* detection, water samples from positive *EC* broth tubes were inoculated into nutrient broths and incubated at 44.5 °C for 24 h to promote *E. coli* growth. The broths were then subjected to the indole test. Colony counting was performed using the pour plate method, visually counting colonies on agar plates to calculate the TVC. Positive coliform detection was indicated by gas production and lactose fermentation within the designated incubation period. Confirmation was achieved by transferring samples to BGBB agar plates and observing the growth of typical *coliform* colonies [75,76,79,[81], [82], [83]].

#### 2.5.13 Description of bacteriological loads of the sample

To determine the bacteriological loads of the sample, the colonies on each plate were counted using a colony counter. The results were described as CFU/ml using the following formula (29).

$$CFU/ml=\frac{number\;of\;colonies\;x\;dilution\;factors}{volume\;of\;the\;sample}$$

### 2.6 Data management and quality control

To ensure data management and maintain quality control throughout the study, a standardized format was implemented for sample collection. This format included essential details such as sample type, location, date and time of collection, collector names, and the number of samples taken. All laboratory materials used for analysis underwent a thorough cleaning process, including washing, rinsing, and sterilization, to preserve sample integrity and eliminate potential contaminants. Qualified professionals, including public health and microbiology experts, were trained in proper sample collection procedures during an orientation session. The collected samples were stored in aseptic conditions in ice chests or chilled cooler bags and transported to the food microbial laboratory at the EPHI. Before analysis, media for sample testing was prepared according to manufacturer instructions, and its sterility was confirmed through overnight incubation. Stringent quality control measures were implemented for each batch of samples to ensure accurate and reliable results.

Tests were run with positive and negative controls for the whole quality of the study under the whole supervision of culture media, reagents and samples were run with positive and negative controls.

### 2.7 Statistical analysis

Descriptive statistics, including frequencies, percentages, and other relevant measures, were used to summarize and analyze the data regarding microorganisms. The collected data were entered into an Excel spreadsheet, and the statistical software STATA version 16 was utilized for the analysis.

## 3 Results

### 3.1 Distribution of food and water samples

**Fig. 1:**
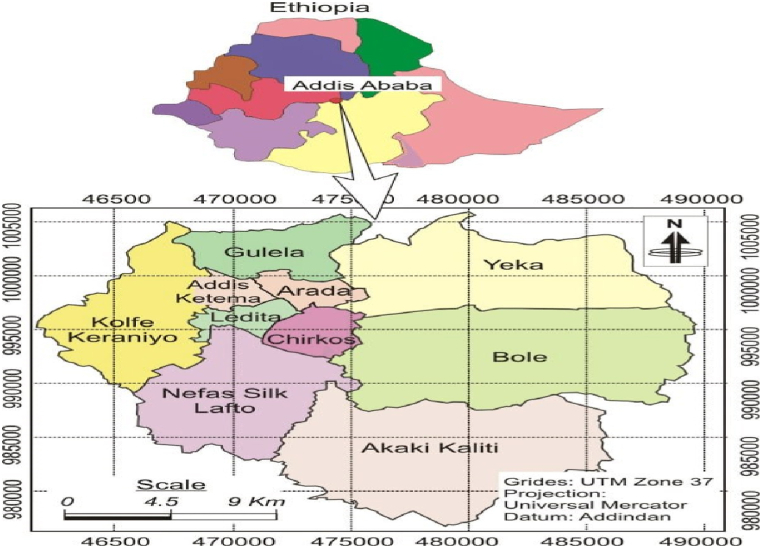
Map and photographs of the study area in Addis Ababa, Ethiopia.

**Fig. 2:**
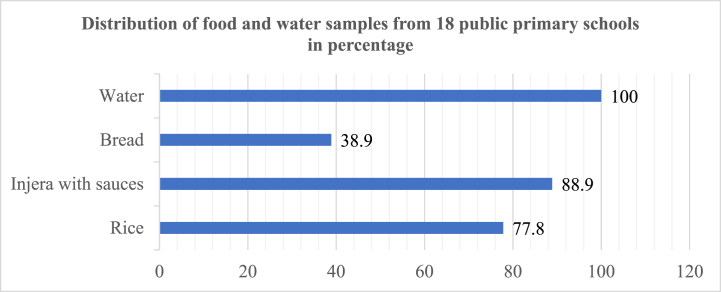
Distribution of food and water samples in the Addis Ababa SFP among 18 public primary schools, Ethiopia (n = 55).

The distribution of samples in this study, as illustrated in Fig. 2, delineates the prevalence of various food items examined Fig. 1. Injera with sauces emerged as the predominant food item, constituting 88.9 % of the total samples, reflecting its ubiquitous presence within the school feeding program. Following closely, rice accounted for 77.8 % of the samples, underscoring its substantial consumption within the sampled schools. Bread, although less prevalent in comparison, still represented a notable portion at 38.9 % of the samples, indicating its significance in the dietary landscape.

Complementing the food samples, water samples from all 18 selected public primary schools were included in the study, encompassing 100 % of the total samples. This comprehensive inclusion highlights the integral role of water quality assessment alongside food safety evaluations, encapsulating a holistic approach to ensuring the well-being of students within the school environment.

### 3.2 Microbiological quality assessment of injera with sauces, rice, and bread served to students

**Fig. 3:**
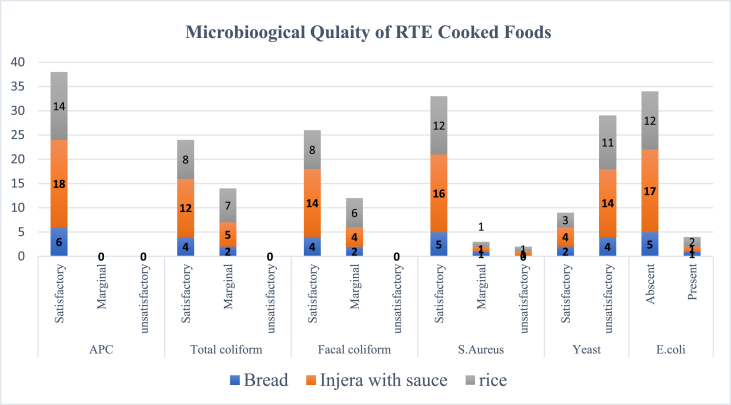
Microbiological assessment of RTE cooked meals served to students in public primary schools as part of the Addis Ababa SFP, Ethiopia (n = 37).

The microbiological quality assessment was conducted on three food items, namely injera with sauces, rice, and bread, which were served to students, and the findings are presented in Fig. 3. The findings revealed important insights into the microbial safety of these food items. In terms of APC, all three food items met the satisfactory criteria. Injera with sauces had a count of 18, rice had a count of 14, and bread had a count of 6. These results indicate that the microbial load in the food items was within acceptable limits and did not pose a significant risk to the students' health.

The evaluation of *TC* bacteria showed that injera with sauces and rice maintained satisfactory levels, with counts of 12 and 8, respectively. However, bread had marginal results with a count of 5. Although the counts in bread were slightly higher than the desired level, they still did not exceed the threshold for unsatisfactory results. Overall, there were no unsatisfactory results for *TC* in any of the food items. Similarly, the assessment of *FC* revealed satisfactory levels in injera with sauces (count of 14) and rice (count of 8), while bread showed marginal levels with a count of 4. Although the marginal result in bread suggests a slightly higher presence of *FC*, it did not reach an unsatisfactory level. None of the food items recorded unsatisfactory results for *FC*.

Yeast and molds, which can impact the quality and shelf life of food, were found to be satisfactory in injera with sauces (count of 4) and rice (count of 3). However, both food items had unsatisfactory results for yeast and molds, with injera with sauces having a count of 14 and rice having a count of 11. Bread also showed marginal results with a count of 2, but no unsatisfactory result was observed. These findings suggest that the food items, particularly injera with sauces and rice, may have had some issues with yeast and mold contamination, requiring attention to maintain their quality. Regarding *S. aureus*, a bacterium associated with foodborne illnesses, injera with sauces, rice, and bread all had satisfactory counts of 16, 12, and 5, respectively. However, there was one marginal result in each food item, with injera with sauces and rice having a count of 1, and bread having a count of 1. Two unsatisfactory results were recorded for *S. aureus* in injera with sauces and rice. These marginal and unsatisfactory results indicate the need for improved hygiene practices during food preparation to prevent the growth of *S. aureus*.

Lastly, the presence of *E. coli*, a common indicator of *FC*, was evaluated. It was found to be absent in injera with sauces, rice, and bread, with counts of 17, 12, and 5, respectively. However, four unsatisfactory results were observed for *E. coli* in rice (two), injera with sauces, and bread (one each), indicating the presence of *E. coli* in four samples. These findings highlight the importance of strict sanitary measures to prevent fecal contamination during the preparation and handling of food items.

### 3.3 Microbial quality of the school meal samples

The examination of the microbial quality of school meal samples involved a comprehensive evaluation of various indicator microorganisms, with outcomes detailed in Table 2. These results offer valuable insights into the microbial content, aiding in the assessment of overall sample safety and quality. Regarding APC, all 37 samples tested fell within the acceptable range, showcasing levels below <10^6^ cfu/ml. This signifies that the microbial content in the samples remained within permissible limits, reflecting a commendable standard of microbial quality.

**Table 2:** Microbial Safety Evaluation of RTE cooked foods samples in the Addis Ababa SFP, Ethiopia (March to April 2024) (n = 37).

Indicator organisms	Microbial quality			
	Food samples examined (n = 37)	Satisfactory	Marginal	Unsatisfactory
		In % and CFU/ml	In % and CFU/ml	In % and CFU/ml
APC	37	100 (<10^6^)	0.0 (10^6^–10^7^)	0.0 (≥10^7^)
*TC*	37	64.9 (<10^2^)	35.1 (10^2^–10^4^)	0.0 (>=^-^10^4^)
FC	37	70.3 (<10^2^)	29.7 (10^2^–10^4^)	0.0 (>=^-^10^4^)
*E. coli*	37	89.5 (<3)	0.0 (3–100)	10.8 (>10^2^)
*S. aureus*	37	89.2 (<10^2^)	5.4 (10^2^–10^3^)	5.4 (10^3^–10^4^)
Yeasts and molds	37	21.6 (<10^2^)	–	78.4 (>10^2^)

In the realm of total coliforms and fecal coliforms, a significant portion of samples (64.9 % and 70.3 % respectively) demonstrated satisfactory levels, maintaining counts below <10^2^ cfu/ml. However, a portion of the samples (35.1 % for *TC* and 29.7 % for FC) fell into the marginal range, indicating a slightly elevated microbial presence. Encouragingly, no samples surpassed the unsatisfactory threshold, underscoring positive strides in ensuring microbial safety.

Evaluation of *E. coli* levels revealed that 89.5 % of samples exhibited satisfactory counts below<3 cfu/ml, with none falling within the marginal range (3–100 cfu/ml). However, it is worth noting that 10.8 % of the samples exceeded the unsatisfactory threshold, indicating a higher presence of *E. coli* in those samples.

Likewise, for *S. aureus,* the majority of samples (89.2 %) displayed satisfactory levels below<10^2^ cfu/ml. A small percentage of samples (5.4 %) resided inthe marginal range (10^2^-10^3^ cfu/ml), and another 5.4 % of the samples were in the unsatisfactory range (10^3^ -10^4^ cfu/ml). These findings underscore the necessity for enhanced control measures to uphold sample microbial safety.

In terms of yeasts and molds, only 21.6 % of the samples had satisfactory levels below <10^2^ cfu/ml. Conversely, the bulk of samples (78.4 %) surpassed the unsatisfactory threshold, signaling a notable presence of yeasts and molds. This outcome suggests potential apprehensions regarding food quality and spoilage, emphasizing the imperative for heightened control measures to curb the proliferation of yeasts and molds in the samples.

### 3.4 Overall microbial quality in school meal samples served in the SFP

**Fig. 4:**
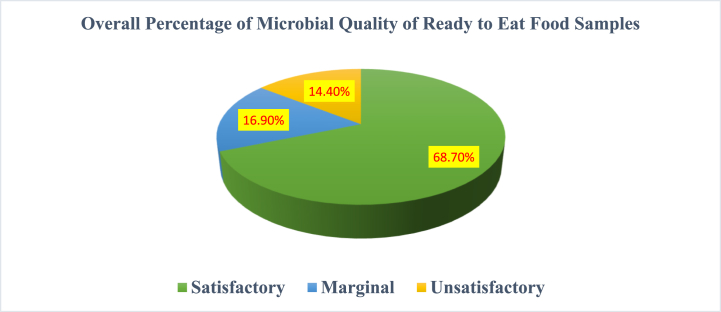
Comprehensive assessment of microbial quality in school meal offerings within the Addis Ababa SFP, Ethiopia (March to April 2024) (n = 37).

In analyzing the overall quality of the samples, a comprehensive assessment was conducted across multiple parameters. The satisfactory category emerged as the dominant classification, encompassing approximately 68.7 % of the samples. Notably, high ratings were observed for the APC (100 %), *TC* (64.9 %), *FC* (70.3 %), *E. coli* (89.5 %), *S. aureus* (89.2 %), and yeasts and molds (21.6 %). Conversely, the marginal category accounted for approximately 16.9 % of the samples, unveiling nuanced concerns primarily in *TC* (35.1 %), *FC* (29.7 %), and *S. aureus* (5.4 %). Within the “unsatisfactory” category, comprising about14.4 % of the samples, specific issues surfaced in parameters such as *E. coli* (10.8 %), *S. aureus* (5.4 %), and yeasts and molds (78.4 %). Referring to the pie chart presented in Fig. 4, the segmented categories provide insights into the distribution of microbial quality levels, offering a detailed understanding of the safety and quality of the school meal samples within the program.

### 3.5 Microbial quality assessment of drinking water samples

Table 3 outlines the findings from the evaluation of 18 drinking water samples designated for student consumption. The analysis focused on key parameters including APC, *TC, FC,* and *E. coli*. In the assessment, 28.8 % met the acceptable limit for APC (<100 cfu/ml), while the majority, constituting 72.2 % of the samples, surpassed this limit (>100 cfu/ml). Concerning total coliforms, 84 % of the samples demonstrated counts below the permissible level (<1 cfu/ml), while 16 % exceeded this threshold (>1 cfu/ml) Table 4.

**Table 3:** Microbial quality assessment of drinking water samples served to students in the Addis Ababa SFP, Ethiopia (March to April 2024) (n = 18).

Indicator organism	Microbial quality of water		
	Drinking water samples (n = 18)	Potable (In % and cfu/ml)	Not potable (In % and cfu/ml)
APC	18	28.8 % (<100)	72.2 % (>100)
*TC*	18	84 % (<1)	16 % (>1)
*FC*	18	94.5 % (<1)	5.5 % (>1)
*E.coli*	18	100 % (<1)	0.0 % (>1)

**Table 4:** Summary statistics of microbial contamination levels in drinking water and food samples within the Addis Ababa SFP, Ethiopia (March to April 2024) (n = 55).

Variable	Obs	Mean	Std. Dev	Min	Max
APC	55	1745.75	2304.902	0	8320
*TC*	55	1437.255	3242.069	8	10000
*FC*	55	371.273	1434.809	5	8500
*S. aureus*	37	196.579	792.411	0	4600
Yeasts and molds	37	2320.541	5129.028	0	30000

*Regarding FC* counts, a small fraction of samples 5.5 % exceeded the recommended limit (>1 cfu/ml), with the vast majority 94.5 % falling below this threshold (<1 cfu/ml). Noteworthy is the absence of *E. coli* in all samples, with every test yielding negative results and all samples remaining well below the stipulated limit (<1 cfu/ml).

#### 3.5.1 Assessment of potability and contamination in drinking water samples

The investigation revealed that a majority of the drinking water samples, approximately 76.6 %, adhered to potable water standards, signifying commendable microbial quality within acceptable limits. Conversely, 23.4 % of the samples exhibited non-potable characteristics pointing to elevated levels of microbial contamination, rendering them unsuitable for consumption.

**Fig. 5:**
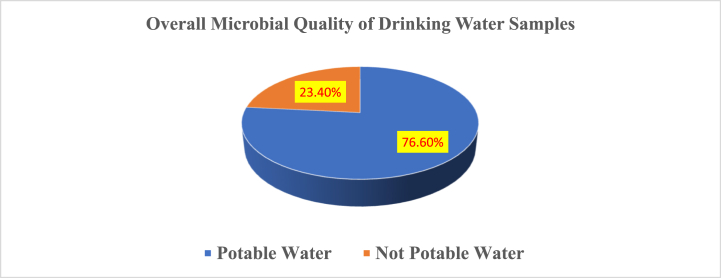
Evaluation of microbial quality in potable and non-potable drinking water samples within public primary schools of the Addis Ababa SFP, Ethiopia (March to April 2024) (n = 18).

The visual depiction in the accompanying pie chart, as illustrated in Fig. 5, succinctly presents these proportions, offering a clear overview of the distribution between potable and non-potable samples. Addressing the issue of non-potable water highlights the crucial imperative to enhance water quality measures, thereby ensuring the provision of safe and clean drinking water for students.

### 3.6 Variability in microbial contamination levels of water and food samples

The examination of microbial contamination levels in water and food samples unveiled substantial variability across multiple parameters, reflecting diverse concentrations present in the samples. Summary statistics offer valuable insights into the breadth and fluctuations in microbial counts within the dataset.

The average count of aerobic microorganisms, captured by the APC metric, stood at 1745.7, indicating a typical concentration observed in the samples. However, the wide range from 0 to 8320 suggests substantial variations in microbial counts among the samples, showcasing notable deviations from the average and underscoring the heterogeneity in contamination levels.

Serving as indicators of water quality, *TC* exhibited an average count of 1437.255. The wide range from 8 to 10000 indicates a significant variation in *TC* concentrations across the samples. This variability underscores the diverse levels of microbial contamination present in the dataset, indicating differences in water quality among the samples.

Specifically utilized to detect fecal contamination in water, *FC* showcased an average count of 371.273. With a high standard deviation of 1434.809 and a range spanning from 5 to 8500, the dataset reveals significant fluctuations in *FC* levels, indicating varying degrees of contamination observed within the samples.

*S. aureus*, a bacterium associated with infections, exhibited an average count of 196.579. The range from 0 to 4600 indicate a considerable variation in the presence of this bacterium across the samples. This finding highlights the need for proper monitoring and control measures to ensure the safety in food samples.

Observations for yeasts and molds revealed an average count of 2320.541. With a wide standard deviation of 5129.028 and a range extending from 0 to 30000, the dataset underscores substantial variability in fungal levels within the samples, underscoring the critical importance of vigilance and management in mitigating fungal contamination in food samples.

## 4 Discussion

The investigation into the microbiological quality of RTE cooked meal samples in the Addis Ababa SFP revealed an overall satisfactory level of quality and safety, aligning with findings from previous studies [84]. However, notable concerns emerged as yeasts and molds exceeded reference standards in 78.4 % of samples (>10^2^ cfu/ml), *E. coli* surpassed limits in 10.8 % of samples (>10^2^ cfu/ml), and *S. aureus* counts exceeded acceptable thresholds in 5.4 % of samples (10^3^ -10^4^), indicating potential risks associated with these pathogens. Cooked rice displayed the highest microbial counts, particularly for *E. coli* and *S. aureus*, corroborating the trend observed in other studies [85]. Approximately 14.4 % of the food samples were deemed unsatisfactory due to contamination from *E. coli, S. aureus,* yeasts, and molds, echoing concerns raised in similar research [86].

Comparing these findings with studies from different regions sheds light on both similarities and differences in RTE food safety practices. For instance, research in Benin City and Bangladesh highlighted similar challenges with microbial contamination in RTE foods, emphasizing the need for improved food safety measures across diverse settings [87,88]. Studies in Bangladesh and India further underscored varying levels of microbial contamination in fast foods, with findings indicating potential health risks posed by pathogens like *E. coli* and Staphylococcus spp [6,25].

The analysis of RTE cooked foods distributed to schools in Argentina and street-vended RTE food in Amravati City, India, revealed comparable concerns regarding microbial contamination, especially concerning pathogens like *E. coli*, and *S. aureus* [84,85]. These findings resonate with the current study's observations and emphasize the widespread nature of microbial risks associated with RTE foods in different contexts.

In the present investigation, *S. aureus* was detected in 5.4 % of food samples, contrasting with studies from Argentina and Brazil where no *S. aureus* was found, highlighting potential variations in microbial contamination levels across regions [50,84]. Similarly, concerning levels of *E. coli* were observed in approximately 10.8 % of samples, aligning with previous reports and emphasizing the critical role of stringent food safety practices in preventing microbial contamination and associated health risks [4,49].

Studies conducted in various regions, such as Gondar, Ethiopia, and the University of Port Harcourt, Nigeria, further underscore the global challenge of bacterial contamination in street-vended foods, with findings revealing the presence of pathogens like *TC*, *S. aureus*, and Salmonella species, reinforcing the need for enhanced food safety measures worldwide [86,89].

Moreover, research on street-vended foods in Jigjiga City, Eastern Ethiopia, highlighted a high contamination rate of 72 % with prevalent isolates being *E. coli and S. aureus,* particularly in foods like ‘Sambusa’ and ‘Pasta’ [4]. A study in Gondar town, Northwest Ethiopia, found that out of 72 street-vended food samples analyzed, 44 were contaminated with *S. aureus and E. coli,* with varying levels of contamination across different food items [90,91]. These findings are consistent with a study in Jimma Town, Southwestern Ethiopia, where *S. aureus* was present in 29.38 % of food samples, predominantly in ‘firfir’, and another study in Gondar, Ethiopia, which reported contamination rates of 64.3 % with pathogenic bacteria, including *E. coli and S. aureus* [92,93].

The presence of even a small number of *E. coli* in food samples is concerning due to the potential for severe foodborne illnesses associated with certain strains [94]. The detection of *E. coli* in RTE foods underscores issues of contamination during food handling and processing, emphasizing the critical need for enhanced hygiene practices and proper cooking techniques to minimize risks.

Our study's findings underscore a significant concern regarding the microbial quality and safety of food samples, particularly in relation to yeasts and molds. A notable proportion of the samples (78.4 %) were deemed unsatisfactory due to elevated levels of these microorganisms. This contamination rate exceeds the results of a comparable study conducted in Burkina Faso, where only 24.75 % of samples exhibited contamination by yeasts and molds [28].

Yeasts and molds serve as indicators of food quality [95], and their presence in food samples not only signifies compromised quality but also poses substantial health risks, especially among school-age children. Molds have the potential to produce mycotoxins, harmful substances with adverse effects on human health. Consequently, immediate attention and remedial action are imperative upon their detection in food samples.

The occurrence of yeasts and molds in heat-treated foods can be attributed to various factors, including inadequate cooking, post-processing contamination, cross-contamination, or the utilization of substandard raw materials. Moreover, the absence of adequate protection from ambient conditions can contribute to food spoilage and the proliferation of these microorganisms.

The assessment of drinking water within the scope of the current school-based study unveiled alarming levels of non-potability, with 23.4 % of samples failing to meet safety standards. Notably, 72 % of the samples exceeded the acceptable APC count (>100 cfu/ml), 16 % surpassed the *TC* limit (>1 cfu/ml), and 5.5 % exceeded the *FC* threshold [84]. This finding resonates with similar studies in Ethiopia and other sub-Saharan regions, where sources such as taps, reservoirs, springs, and wells commonly exhibit microbial contaminants, indicating a pervasive issue that transcends individual study locations [35,37]. Investigations in diverse regions have shed light on the challenges surrounding drinking water quality. For instance, research in the rural villages of the Mohale Basin in Lesotho revealed high levels of pollution in drinking water, with *E. coli* detected in 78 % of unprotected water sources and 60 % of protected sources, alongside signs of open defecation in 59 % of samples, underscoring sanitation and hygiene challenges in such settings [37,39].

In the current study, the analysis of drinking water samples highlighted substantial contamination levels, with notable percentages testing positive for *APC, TC*, and *FC*, indicative of unsatisfactory conditions [84]. This contrasts with findings from studies in Argentina and Italy, where lower levels of contamination were reported, emphasizing regional disparities in water quality and contamination sources [51,84].

Regional variations in water quality were further underscored by research findings from Peshawar, Jaipur, Karnataka, Bhilai, and Tabuk, indicating varying percentages of drinking water samples contaminated with coliforms [[96], [97], [98]]. These discrepancies emphasize the necessity for tailored assessments and targeted interventions to address microbial contamination effectively.

The absence of *E. coli* in the drinking water samples from the current study aligns with findings from Argentina but differs from reports in Assam, India, Peshawar, Pakistan, and Bo, Sierra Leone, where substantial proportions of samples were contaminated with *E. coli* [84,96,99,100]. Compliance with WHO guidelines stipulating non-detectable levels of *TC* bacteria in 100 ml samples further underscores the critical need for stringent water quality standards [37].

Comparing research outcomes from different regions, such as Saudi Arabia, India, Pakistan, and Sierra Leone, reveals varying contamination rates with *coliforms* and *E. coli* in drinking water samples, highlighting global challenges with microbial contamination in water sources [96,[99], [100], [101]]. Studies focusing on municipal drinking water quality in Addis Ababa City, Ethiopia, and other regions within the country underscore the presence of bacterial indicators in public taps, protected wells, and water lines, reflecting the broader concern of water quality and safety [37,[40], [41], [42]].

Challenges in ensuring the quality and safety of drinking water persist due to contaminants stemming from both man-made and natural sources. Microorganisms present in drinking water, often due to fecal contamination and microbial growth within distribution systems, pose significant public health risks, underscoring the importance of robust water quality management practices [37,43]. It is critical to acknowledge the constraints inherent in this study. Initially, the focus on indicator microorganisms serves to highlight unsanitary conditions or potential health hazards. However, the mere presence of these indicators does not confirm the existence of specific pathogens, necessitating further inquiry for precise identification and quantification. Moreover, the study's narrow focus on a limited number of schools may impede the extrapolation of results to encompass all public primary schools in the city. The research underscores the significance of ongoing surveillance to safeguard consumers against foodborne illnesses and food poisoning by ensuring the microbiological safety of RTE foods. This is particularly vital for the well-being of primary school students, who may not always prioritize their dietary choices. While emphasizing the need for nutritious and safe food, the study's limitations, such as the incomplete characterization of all bacterial and fungal isolates, could potentially underestimate the actual extent of food contamination. Additionally, the absence of investigations into parasites, and the practices of food handlers hampers a comprehensive understanding of the factors influencing the microbiological quality of RTE foods.

## 5 Conclusions

The findings underscore the pressing need for immediate action to address microbial safety challenges within the Addis Ababa SFPs. While most samples adhered to acceptable standards, the presence of heightened levels of *E. coli, S. aureus*, and yeasts/molds emphasizes critical areas of concern. Elevating hygiene standards during food preparation and enforcing stringent control measures are paramount to ensure student safety and well-being.

Moreover, comprehensive interventions are essential to combat microbial contaminants in drinking water, mandating improved hygiene practices, safeguarding water sources, and implementing regular monitoring protocols. Prioritizing the microbial safety of food and water is indispensable for effectively safeguarding the health and welfare of students enrolled in the Addis Ababa SFP. The novel findings presented in this study not only highlight the existing challenges but also underscore the urgency and importance of implementing proactive measures to enhance food and water safety protocols within SFP. This study contributes valuable insights that can guide future research and policy initiatives aimed at ensuring the optimal health outcomes for school children.

## Funding statement

This work was supported by the 10.13039/501100000193IDRC through the “Urban agriculture for advancing healthy and sustainable food systems in Addis Ababa” project.

## Data availability

The data related to this study has not been deposited in a publicly available repository; however, it will be accessible upon request.

## Ethical approval

The IRB of the College of Natural and Computational Sciences, AAU, with Ref. No. CNCSDO/623/15/2023. Additionally, permission was obtained from the AAEB. Prior to the collection of food and water samples, the directors of all selected primary schools provided their consent. The confidentiality of all sample sources was maintained by unique identification code.

## CRediT authorship contribution statement

**Yihalem Tamiru:** Writing – review & editing, Writing – original draft, Visualization, Validation, Software, Resources, Project administration, Methodology, Investigation, Funding acquisition, Formal analysis, Data curation, Conceptualization. **Abebe Ayelign:** Writing – review & editing, Visualization, Validation, Supervision, Project administration. **Afework Mulugeta:** Writing – review & editing, Visualization, Validation, Supervision, Project administration, Methodology. **Samson Gebremedhin:** Writing – review & editing, Visualization, Validation, Supervision, Resources, Project administration, Methodology, Funding acquisition.

## Declaration of competing interest

The authors declare the following financial interests/personal relationships which may be considered as potential competing interests:YIHALEM TAMIRU reports financial support was provided by International Development Research Center (10.13039/501100000193IDRC). If there are other authors, they declare that they have no known competing financial interests or personal relationships that could have appeared to influence the work reported in this paper.
